# DASH Diet: A Review of Its Scientifically Proven Hypertension Reduction and Health Benefits

**DOI:** 10.7759/cureus.44692

**Published:** 2023-09-04

**Authors:** Chidera Onwuzo, John o Olukorode, Olutomiwa A Omokore, Oluwatobi S Odunaike, Raymond Omiko, Osadebamwen w Osaghae, Walid Sange, Dolapo A Orimoloye, Heritage O Kristilere, Ehizobhen Addeh, Somtochukwu Onwuzo, Lisa Omoragbon

**Affiliations:** 1 Internal Medicine, Benjamin S. Carson (Snr) College of Health and Medical Sciences, Ilishan-Remo, NGA; 2 Internal Medicine, General Hospital Lagos Island, Lagos, NGA; 3 Internal Medicine, K. J. Somaiya Medical College and Research Centre, Mumbai, IND; 4 Internal Medicine, College of Medicine University of Lagos, Lagos, NGA; 5 Internal Medicine, Cleveland Clinic Foundation, Cleveland, USA

**Keywords:** benefit of dash diet, approach to dash diet, clinical trials, dash diet, hypertension

## Abstract

The Dietary Approach to Stop Hypertension (DASH) constitutes a nonpharmacological dietary strategy tailored with the primary objective of mitigating hypertension and averting its potential complications. Numerous clinical studies, such as the PREMIER trial, DASH sodium study, and OmniHeart trial, as well as other studies, substantiate the DASH diet's ability to manage hypertension. Beyond its profound impact on hypertension reduction, the DASH diet has exhibited notable efficacy in addressing an array of conditions such as heart failure, lipid homeostasis, dyslipidemia, and uric acid dysregulation. With its empirical foundation, the DASH diet emerges as an indispensable tool in the hypertension management toolkit, warranting its exploration and integration into various medical contexts.

This review commences with an overview of both the DASH diet and the significance of hypertension as a prevailing health concern. The ensuing discussion meticulously examines the extensive body of clinical research, firmly establishing the DASH diet's prowess in hypertension management. Furthermore, this review delves into the strategic approaches necessary for the successful implementation of the DASH diet, outlining the roles of technology and governmental responsibilities in ensuring its widespread adoption. As a comprehensive examination of the DASH diet's efficacy and potential, this review underscores its significance in modern healthcare paradigms.

## Introduction and background

The Dietary Approach to Stop Hypertension (DASH) diet, originally formulated by the National Institutes of Health (NIH), highlights a comprehensive intake of nutrient-rich foods. Aligned with heart-healthy guidelines, this dietary approach restricts saturated fat and cholesterol consumption. A central tenet of the diet involves enhancing the intake of nutrient-dense foods recognized for their influence on reducing blood pressure. These foods are typically high in minerals such as potassium, calcium, and magnesium, as well as protein and dietary fiber. Notably, the DASH diet is designed to encompass a food spectrum that aligns with the nutritional guidelines recommended by the Institute of Medicine [1].

With its foundation rooted in evidence-based research, the DASH diet encourages the intake of fruits, vegetables, whole grains, lean proteins, and low-fat dairy products while reducing sodium, sugary beverages, and processed foods. By adhering to these dietary principles, individuals are encouraged to achieve and maintain optimal blood pressure levels [1].

Hypertension as a health concern

According to the guidelines provided by the American College of Cardiology (ACC) and the American Heart Association (AHA), hypertension is diagnosed when blood pressure consistently measures ≥130 or ≥80 mmHg [3]. Approximately one in three American adults is hypertensive, consequently earning it the ominous label of *the silent killer* due to its tendency to often manifest without obvious symptoms until complications like heart disease, stroke, kidney disease, and even vision impairment arise [2].

## Review

Scientific evidence of the benefit of the DASH diet in hypertension management

The DASH diet has emerged as a prominent dietary strategy for managing hypertension and promoting cardiovascular health. These pieces of scientific evidence serve to substantiate this claim.

The PREMIER trial investigated the effects of lifestyle interventions, including the DASH diet, on blood pressure reduction. This landmark study involved 810 participants with prehypertension (120-139/80-89 mmHg) and stage 1 hypertension (140-149/90-95 mmHg). The participants were assigned to different groups: the *Advice only* group, the established group (consisting of weight loss, increased physical activity, and reduced sodium and alcohol intake), and the established plus DASH diet group. Findings showed a decrease in the systolic blood pressure of 6.6 mmHg in the Advice only group, 10.1 mmHg in the established group, and 11.1 mmHg in the established plus DASH diet group [4].

The DASH-Sodium trial specifically examined the impact of sodium intake in combination with the DASH diet on blood pressure. This trial included three groups: a control group on a typical American diet, a group on the DASH diet with higher sodium intake, and a group on the DASH diet with lower sodium intake. The results revealed that the DASH diet alone led to a significant reduction in blood pressure. However, when combined with sodium reduction, the blood pressure reduction was even greater. Participants following the DASH diet with low sodium intake experienced an average systolic blood pressure reduction of 7.1 mmHg in those without hypertension and 11.5 mmHg in those with hypertension [5].

The OmniHeart trial aimed to evaluate the effects of three different diets, including a variation of the DASH diet, on blood pressure and cardiovascular risk factors. This trial explored the impact of a protein, unsaturated fats, and carbohydrate-rich diet on participants with hypertension or prehypertension. Results indicated that all three diets contributed to improved blood pressure levels with greater reduction seen with the modified DASH diet than the DASH diet alone [6].

Saneei et al. conducted a systematic review and random effects meta-analysis to evaluate the impact of the DASH diet on blood pressure. Their study encompassed 17 randomized controlled trials (RCTs) involving 2,561 participants. The results of their meta-analysis indicated a statistically significant reduction in systolic blood pressure by 6.74 mmHg and diastolic blood pressure by 3.54 mmHg. Moreover, subgroup analysis revealed that RCTs incorporating energy restriction and hypertensive subjects exhibited more pronounced reductions in blood pressure. Additionally, baseline blood pressure levels were identified as significant contributors to inter-study variance. These findings show the potential of the DASH diet in reducing blood pressure, albeit with varying degrees contingent upon factors such as energy intake and participants' initial blood pressure levels [7].

Blumenthal et al. elucidated the combined effects of the DASH diet, exercise, and weight loss on blood pressure and cardiovascular biomarkers. Participants in the ENCORE study were overweight individuals with above-normal blood pressure. The study involved three groups: DASH diet alone, DASH diet with behavioral weight management, and usual diet control. Clinic-measured blood pressure changes were substantial in the active treatment groups, with the DASH diet combined with weight management showing the most significant reductions. Adjusted changes in systolic blood pressure were 16.1, 11.2, and 3.4 mmHg for the respective groups. Importantly, the combined approach demonstrated improvements in vascular and autonomic functions, alongside a reduction in left ventricular mass, underscoring the added benefits of incorporating exercise and weight management with the DASH diet for overweight individuals with high blood pressure [8].

Furthermore, a study published in the *American Heart Association Journal* in 2001 investigated the efficacy of the DASH diet in treating Stage 1 Isolated Systolic Hypertension (ISH). Among 459 participants from the DASH trial, 72 individuals with ISH were identified. During the intervention period, the DASH diet group experienced a significant decrease in systolic blood pressure by 11.8 ± 9.3 mmHg and a notable reduction in diastolic blood pressure. While urinary sodium levels remained consistent across groups, urinary potassium levels increased in the fruits/vegetables and DASH diet groups [9].

Other health benefits of the DASH diet

Beyond its renowned efficacy in reducing blood pressure, emerging evidence has unveiled a broader spectrum of health benefits associated with the DASH diet. It has exhibited discernible effects on lipid profiles, resulting in reductions in low-density lipoprotein (LDL) and triglyceride concentrations [10]. While improvements in high-density lipoprotein (HDL) and total cholesterol were not statistically significant, the diet showcased a reduction of approximately 13% in the estimated 10-year risk of cardiovascular disease [11].

Moreover, adopting the DASH dietary pattern has been associated with a diminished incidence of heart failure in individuals under 75 years of age [12], alongside a decrease in the prevalence of heart failure-related hospitalization and mortality in men [13].

The amalgamation of the DASH diet and reduced sodium intake has exhibited complementary effects on decreasing bone turnover, leading to improved bone mineral status. This effect was observed through reductions in serum osteocalcin, C-terminal telopeptide of type 1 collagen, serum parathyroid hormone (PTH) levels, and urinary calcium [14].

The DASH diet extends to the reduction of uric acid levels. An RCT by Tang et al. showcased that the introduction of the DASH diet led to notable decreases in uric acid levels at both 30 and 90 days. This implies its potential recommendation for patients with hyperuricemia or gout [15]. Furthermore, multiple studies have consistently demonstrated the DASH diet's association with lower all-cause mortality rates [16].

Approaches for effective DASH implementation

The significance of the DASH diet in managing hypertension and overall health is undeniable. It’s crucial to recognize that effectively implementing the DASH diet goes beyond theoretical knowledge. It necessitates a practical approach encompassing early presentations, counseling, technological integration, and government support. These recommendations would be discussed under the following sections.

Early Presentation and Assessment

**Figure 1:**
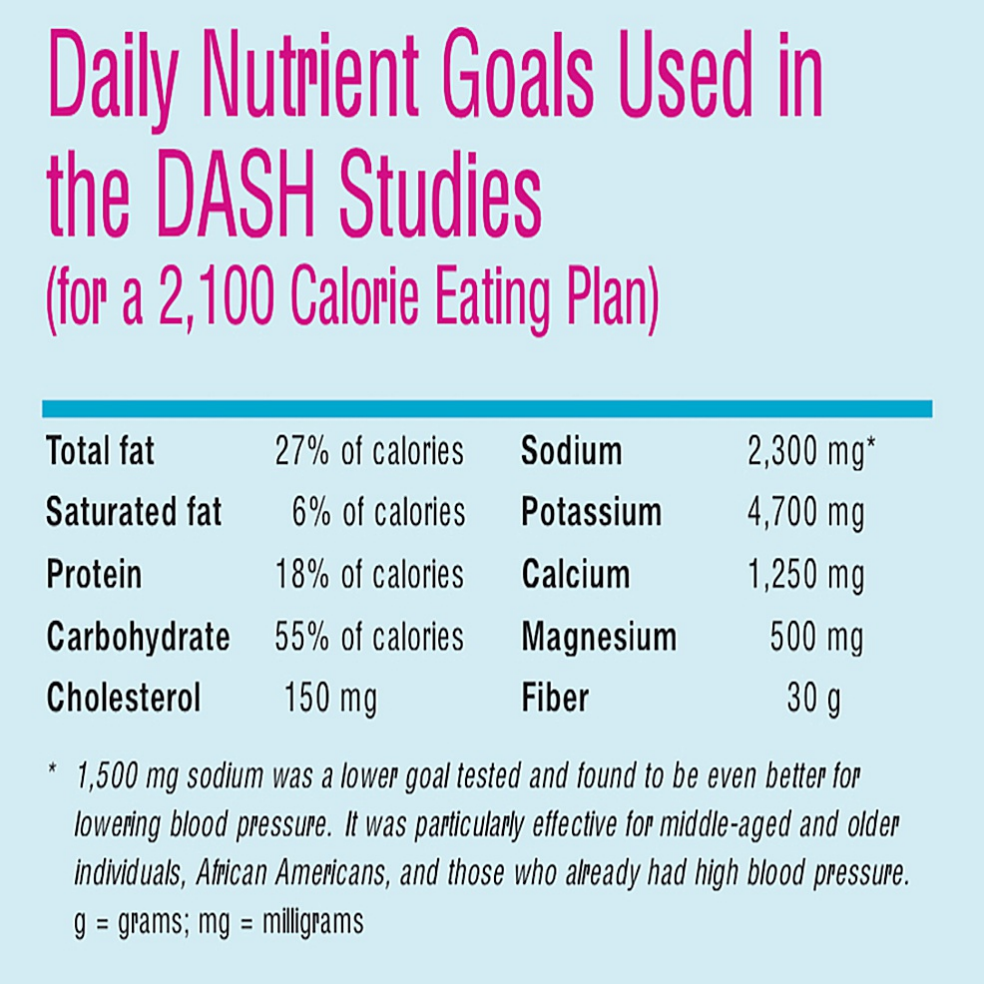
The daily nutrient goals for a 2,100-calorie eating plan used in the DASH studies. Source: [1]. DASH, Dietary Approach to Stop Hypertension

**Figure 2:**
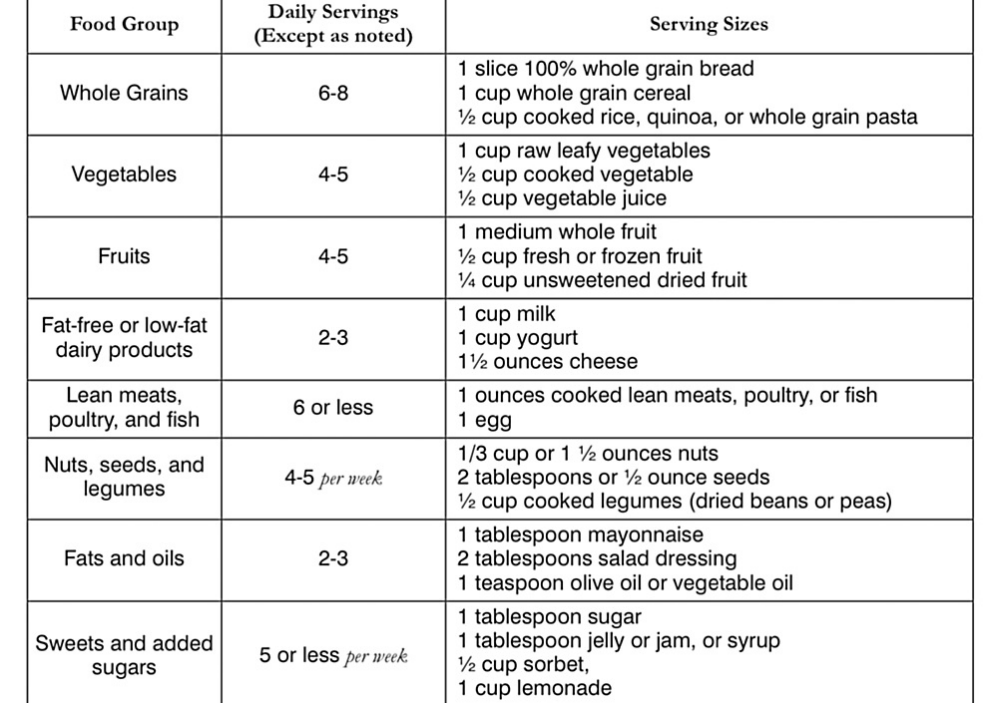
A typical example of a DASH eating plan. Source: [2]. DASH, Dietary Approach to Stop Hypertension

Practical considerations for implementing the DASH diet begin with the initial step of referring the patient to a registered dietitian for an assessment of their suitability for the DASH diet plan. Subsequent steps involve calculating the individual's caloric requirements and engaging in a detailed discussion about meal planning benefits, setting weight goals, and utilizing shopping lists to achieve established targets. Further enlightenment on healthy cooking habits and possible enrollment in such classes would be beneficial (Figures 1-2) [17].

Counseling and Education

To further enhance the successful implementation of the DASH dietary plan for hypertensive adults, it becomes imperative to incorporate effective counseling and education. The United States Preventive Services Task Force classifies counseling into brief, medium-intensity, and high-intensity sessions. Brief sessions, typically around five minutes during medical visits, propose achievable lifestyle changes. Medium-intensity sessions, lasting at least 30 minutes, involve group or individual discussions, guided by trained dietitians and primary care experts. These sessions encompass focused group discussions, motivational interviewing, and motivational counseling. Taking intensity a step further, High-Intensity Sessions extend over up to six years, delivering a profound impact. Noteworthy examples include organizing workshops, seminars, and retreats, supplemented by vital follow-ups [18]. 

Technology Integration

In our society today, the impact of technology and digital revolution cannot be overstated in every aspect of life. As such, a recent study outlines the innovation of technology in the application of the DASH diet. It describes a system with relevant factors considered due to recommendations for the dietary plan. A mobile application with data storage allows people to engage with user interface software and input data into the system which then generates recommendations using DASH dietary guidelines. The convenience of use in mobile phones, as they are integral to everyday life, makes it practical. Cloud-based database systems are used for storage and authentication. The incorporation of user profiles and the development of a DASH Diet Food Database are additional solutions made possible through technological innovation [19].

Governmental Support

Governmental policies have also been helpful to the propagation of the DASH diet in places where they are optimally active. One of such priority policies that have been advocated for in the DASH studies is the availability of its constituent components across multiple retail outlets and grocery stores. In addition, it is consistent with many global recommendations on healthy and optimal living, which were declared by several organizations that are world leaders. Some of these recommendations include the Dietary Guidelines for Americans, the National Cancer Institute, and the National Cholesterol Education Program’s Step 2 Diet [20].

## Conclusions

The DASH diet has been scientifically proven to be an antique but trenchant tool in the armamentarium used in the fight against hypertension. Collaterally, it is beneficial in lipid regulation, heart failure, bone health, and uric acid homeostasis. Furthermore, its use in synergy with other pharmacological methods can lead to even more profound results. We recommend that all general practitioners, internal medicine specialists, dietitians, and government agencies incorporate the DASH diet into their management of hypertension and policymaking while employing a practical approach.
